# Electrochemical reduction of unsaturated carbon–carbon bonds via 3d transition-metal catalysis

**DOI:** 10.3762/bjoc.22.75

**Published:** 2026-06-17

**Authors:** Geon Kang, Minki Jeon, Pooja Kumari Jat, Cheoljae Kim, Isaac Choi

**Affiliations:** 1 Department of Chemistry, Chungbuk National University, Chungcheongbuk-do, 28644, Republic of Korea

**Keywords:** electrochemical reduction, electroorganic synthesis, reaction mechanism, 3d transition metals, unsaturated C–C bonds

## Abstract

Electrochemical reduction has emerged as a powerful alternative to conventional hydrogenation for unsaturated C–C bonds, enabling precise control without molecular hydrogen or stoichiometric reductants. This review summarizes recent advances in iron-, cobalt-, and nickel-catalyzed electroreduction of alkynes and alkenes, highlighting how electrochemical parameters and catalyst design unlock distinct, controllable reaction manifolds that are inaccessible under thermochemical conditions. These developments position 3d metal electrocatalysis as a versatile and programmable platform for selective hydrogenation and isotopic labeling under mild, sustainable conditions.

## Introduction

### Reductions of unsaturated C–C bonds

Reduction of unsaturated C–C bonds, most notably, alkynes and alkenes, is a synthetic method that has been widely and extensively discussed with a long and rich history among chemistry and chemical industry community [1–9]. Given this longstanding significance, the development of controlled reduction strategies has diversified to accommodate the distinct reactivity and selectivity. Representatively, complete hydrogenation of alkynes or alkenes is conventionally achieved by exposing substrates to molecular hydrogen in the presence of homogeneous transition-metal catalysts or supported catalysts, thereby affording the corresponding alkanes. In sharp contrast, when selective reduction of alkynes is required, the use of Lindlar’s catalyst provides a controlled *syn*-addition pathway that furnishes *Z*-alkenes [10], whereas Birch-type reductions via single-electron transfer (SET) promote an *anti*-addition process that enables the formation of *E*-alkenes [11–12].

Beyond the classical strategies, recent advances have enabled the highly efficient chemo- and stereoselective hydrogenation of alkynes under electrochemical and transition-metal-catalyzed conditions. The alkyne semihydrogenation generally benefits from the stronger coordination ability of the C–C triple bonds and the greater accessibility of metal-bound intermediates, thereby often enabling stereocontrol through inner-sphere substrate organization. From this perspective, alkene reduction is relatively more challenging because of the lower π-basicity of alkenes. Alternatively, outer-sphere electron transfer, mediated by electrolysis, or the strategic use of redox mediators and co-catalysts could also provide effective approaches for promoting selective alkene hydrogenation.

### Electrochemical and 3d transition-metal catalysis

Owing to the straightforwardness of the reductive chemical transformation, a plethora of synthetic approaches has been utilized, with recent efforts increasingly converging on 3d transition-metal catalysis [13–18] that enables cost-effective hydrogenation reactivity and electroorganic synthetic strategies [19–37] that employ externally applied potentials to achieve bond reduction with operational sustainability. First of all, the recent emergence of homogeneous 3d transition-metal catalysis has reshaped reductive methodologies, as these earth-abundant metals offer accessible redox manifolds and characteristic SET behavior that directly address the economic and environmental constraints of noble-metal-based systems [38–39]. In sharp contrast, electroorganic synthesis provides a fundamentally distinct mode of reactivity compared to conventional thermochemical approaches, in which electric current serves as the terminal reductant, thereby enabling unconventional mechanistic pathways for alkyne reduction while circumventing the need for molecular hydrogen or stoichiometric reducing agents. Consequently, the merger of 3d transition-metal catalysis with electroorganic synthesis represents a conceptual shift beyond traditional reagent-driven approaches, enabling precise control over redox events through the applied potential while expanding access to fundamentally distinct mechanistic manifolds [40]. In particular, this electrochemical toolbox enables catalyst-controlled engagement of reaction pathways that are difficult to access under thermochemical conditions, thereby broadening the scope of achievable reactivity and selectivity [29].

#### A design for electroorganic synthesis

**Figure 1:**
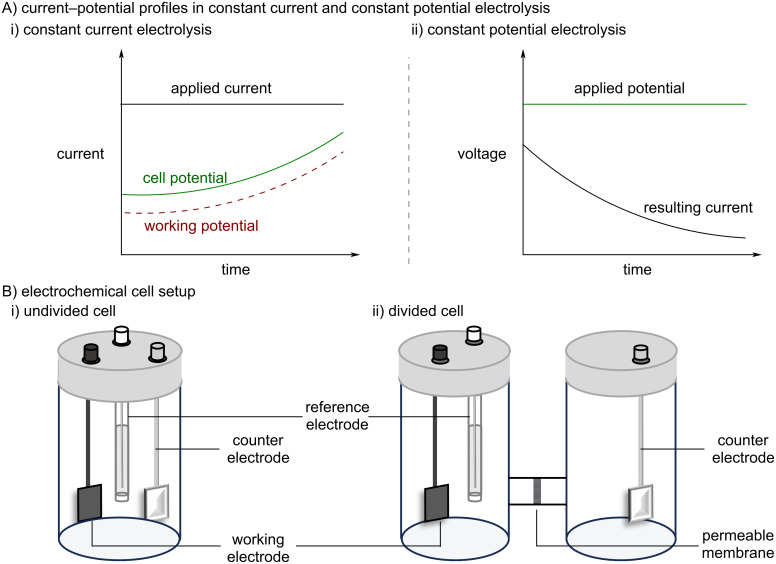
A design for electroorganic synthesis.

Chemical transformations are inherently governed by the movement of electrons. Consequently, electrochemistry offers a platform in which an applied potential directly controls the electron flow, enable redox processes without the use of stoichiometric reagents. In this context, when electrochemistry is employed as a synthetic tool in organic chemistry, several critical parameters must be taken into account, including (a) electrode, (b) electrolysis mode, and (c) cell configuration (Figure 1).

Among these, the choice of electrode is particularly critical, as it directly governs interfacial electron transfer [41–45]. In most preparative settings, a two-electrode system is adopted for its operational simplicity, wherein the anode withdraws electrons and the cathode delivers them, thus maintaining charge balance. As this review primarily addresses reductive electroorganic transformations, the anodic process is often not directly involved in product formation. In such cases, sacrificial anodes, most commonly zinc or magnesium, are employed, wherein the electrode material undergoes dissolution to maintain charge balance. In certain instances, the resulting metal ions may also participate directly in the reaction process. Notably, since electron transfer occurs at the electrode interface, the nature of the electrode material can directly influence both reactivity and selectivity. Nevertheless, such choices are still largely guided by empirical precedent rather than predictive understanding.

Secondly, the choice of electrolysis mode is of equal importance (Figure 1A) [46–48]. Constant current electrolysis (CCE) is widely employed due to its simplicity and scalability, with the total charge serving as a convenient parameter for reaction control. In sharp contrast, constant potential electrolysis (CPE) enables selective activation of specific redox events by maintaining a defined electrode potential, thereby allowing precise control over electron transfer and minimizing undesired processes. Finally, the configuration of the electrochemical cell further modulates reaction outcomes (Figure 1B). While undivided cells are generally preferred for their simplicity, as both electrodes operate within a single compartment, divided cells are required when anodic and cathodic processes must be spatially separated to prevent undesired interactions between reactive intermediates. Although such separation improves selectivity and mechanistic control, it may also introduce increased cell resistance and mass transport limitations. Consequently, the choice of cell configuration is closely tied to the stability of intermediates and the compatibility of the anodic and cathodic processes. In this review, the distinction between these two cell configurations is represented schematically by the number of bars placed between the electrodes, where a single bar denotes an undivided cell and a double bar denotes a divided cell.

#### Modes for the reduction of C–C bonds

**Figure 2:**
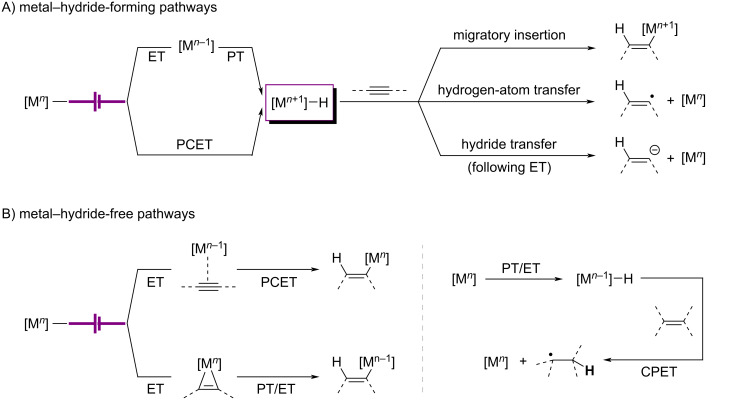
Modes of transition-metal-catalyzed electrochemical reduction of unsaturated C–C bonds.

Electrochemical reduction of unsaturated C–C bonds is dictated by the mode of electron and proton delivery, and the identity of the metal catalyst or redox mediator (Figure 2). Within this framework, the underlying mechanistic modes for the reduction of unsaturated C–C bonds can be broadly classified according to whether the reaction proceeds through a discrete metal hydride (M–H) intermediate (Figure 2A) [49–53] or through a hydride-free pathway (Figure 2B).

In the first category, electrochemical reduction/protonation sequences, or PCET-enabled hydride generation provide catalytically relevant M–H species, which subsequently engage with unsaturated substrates through distinct reaction modes. One prominent mode is migratory insertion, in which the coordinated alkene or alkyne inserts into the M–H bond to form a metal–alkyl or metal–alkenyl intermediate. In some cases, this organometallic species can further undergo chain walking, where iterative β-hydride elimination and reinsertion enable migration of the metal center along the carbon skeleton before product release, thereby allowing remote hydrogenation or positional selectivity [54–56]. The second mode within M–H chemistry is hydrogen-atom transfer (HAT), namely MHAT [38,57–63], in which the electrogenerated M–H intermediate behaves not as a classical two-electron hydride donor but as a one-electron H-atom donor. In such cases, reactivity is governed primarily by bond dissociation free energies, radical stabilization, and cage escape or recombination kinetics, rather than by inner-sphere insertion chemistry. In addition, in certain systems, hydride transfer can operate as a distinct mechanistic mode, wherein the M–H unit directly delivers a hydride equivalent to the substrate, followed by protonation to furnish the reduced product [64].

In sharp contrast, the second major category comprises hydride-free pathways, in which hydrogenation is achieved without the involvement of a discrete M–H intermediate. These mechanisms arise either from direct concerted proton–electron transfer (CPET) to the substrate or from substrate binding to electrochemically generated low-valent metal centers prior to protonation, in some cases, leading to metallacyclic intermediates. Subsequent reduction then proceeds through stepwise ET/PT sequences or PCET processes [65–67], to deliver the formal equivalents of hydrogen. Notably, in certain systems, molecular mediators can directly deliver hydrogen atom equivalents to the substrate via CPET, thereby bypassing the need for metal hydride intermediates altogether and enabling efficient hydrogenation through radical-type pathways. Mechanistically, these pathways depart from the classical two-electron noble-metal hydrogenation paradigm and instead operate through low-valent or electronically flexible intermediates, often involving single-electron or electronically asynchronous reactivity, enabled by the accessible redox states and ligand-dependent electronic flexibility of earth-abundant transition metals. Consequently, modern electrochemical hydrogenation of unsaturated C–C bonds is understood not as a single mechanistic class, but rather as a continuum encompassing diverse reaction manifolds.

Within this mechanistic landscape, 3d transition-metal catalysts occupy a uniquely versatile position, as their accessible redox states and ligand-dependent electronic tunability enable engagement with both metal hydride-involved and hydride-free pathways. Consequently, electrochemical control over electron and proton delivery allows these systems to access various distinct mechanistic pathways, thereby enabling reactivity and selectivity patterns that are not readily attainable under conventional thermochemical conditions.

Beyond batch electrolysis setups, flow cells [68–74] and zero-gap configurations [75–78] have recently emerged as promising platforms for scalable electrochemical hydrogenation reactions. These advanced architectures offer enhanced mass transport, significantly reduced ohmic resistance, and the capability to operate at industrially relevant current densities, thereby improving the efficiency and practicality of unsaturated C–C bond hydrogenation processes.

This review explores recent advances in the electrochemical reduction of unsaturated C–C bonds mediated by 3d transition metals, with a particular focus on iron, cobalt, and nickel-based molecular catalysts, while mechanistic design principles are particularly highlighted. By correlating catalyst structure, electrochemical behavior, and reaction outcome, this review aims to provide a unified framework for understanding how electrochemical control enables new reactivity modes in 3d metal-catalyzed hydrogenation chemistry, while offering perspectives on future directions and remaining challenges in this rapidly developing field.

## Review

### Iron

#### Alkynes to alkenes

Despite the abundance and rich redox chemistry of iron [79–80], as well as its well-established role as a molecular electrocatalyst for small molecule activation [81–86], the application of iron catalysis to the electroreductive transformation of unsaturated C–C bonds remains relatively underdeveloped. This limitation arises primarily from the intrinsic difficulty in stabilizing low-valent iron species under strongly reducing electrochemical conditions, coupled with the propensity of such intermediates to promote undesired hydrogen evolution in the presence of proton sources.

**Scheme 1:**
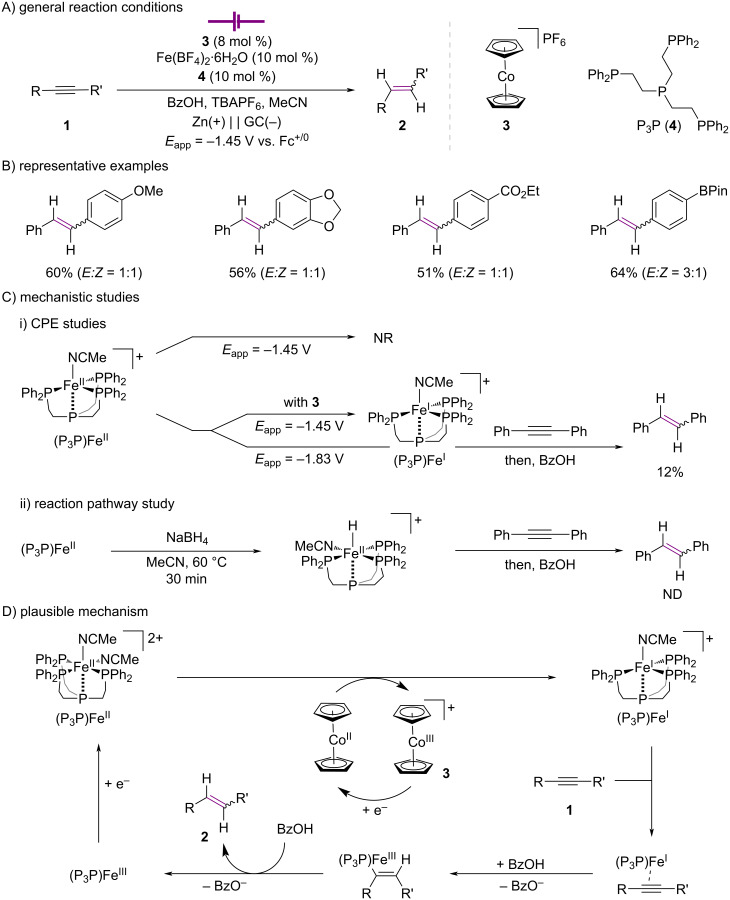
Electroreductive iron catalysis for alkyne semihydrogenation enabled by a cobaltocene redox mediator.

In 2025, the Derosa group systematically evaluated the feasibility of electroreductive iron catalysis using alkyne semihydrogenation as a model transformation under controlled potential electrolysis in an undivided cell setup (Scheme 1A) [87]. Notably, this study demonstrates a rare and conceptually important strategy to selectively access low-valent Fe(I) intermediates under electrochemical conditions via a redox mediator, thereby overcoming the challenge of uncontrolled overreduction in iron electrocatalysis. The reaction proceeds efficiently with internal diarylalkynes bearing both electron-donating and electron-withdrawing substituents, while functional groups typically sensitive to reductive conditions, such as aryl halides and arylboronic esters, are well tolerated (Scheme 1B). Cyclic voltammetric studies revealed distinct Fe(II/I) and Fe(I/0) redox events for (P_3_P)Fe(II)(BF_4_)_2_. Notably, introduction of cobaltocene (Cp_2_Co) as a redox mediator enabled selective SET at a more anodic potential, thereby suppressing competitive hydrogen evolution and rendering tandem electrocatalysis essential for productive reactivity.

During controlled-potential electrolysis, a characteristic color change of the solution was observed, and formation of an electrogenerated Fe(I) species was unambiguously confirmed by X-ray diffraction analysis and UV–vis spectroscopy in the presence of Cp_2_Co, indicating that Fe(I) is not merely a transient species but is accumulated under the operating conditions via a mediator-driven uphill electron transfer. Importantly, this Fe(I) species was shown to be catalytically competent, as exposure to tolane and benzoic acid resulted in the formation of stilbene (Scheme 1C, i). These observations support a tandem electrocatalytic cycle involving sequential Cp_2_Co-mediated reduction of Fe(II) to Fe(I), followed by substrate engagement and regeneration of Fe(II) via a transient Fe(III) intermediate, thus overall implicating a direct substrate coordination to the low-valent iron species rather than proton-first pathways.

In sharp contrast, stoichiometric experiments revealed that an independently prepared (P_3_P)Fe(II)–H species is largely unreactive toward internal alkynes (Scheme 1C, ii). Collectively, these findings strongly favor a hydride-free reaction pathway [88], in which a reduced Fe(I) species mediates alkyne semihydrogenation through net reductive protonation rather than classical metal hydride insertion pathways.

#### Alkenes to alkanes

In sharp contrast to the reduction of alkynes, the direct electrochemical reduction of unactivated alkenes is intrinsically challenging due to their negative reduction potentials, which often preclude selective substrate reduction under synthetically practical conditions.

**Scheme 2:**
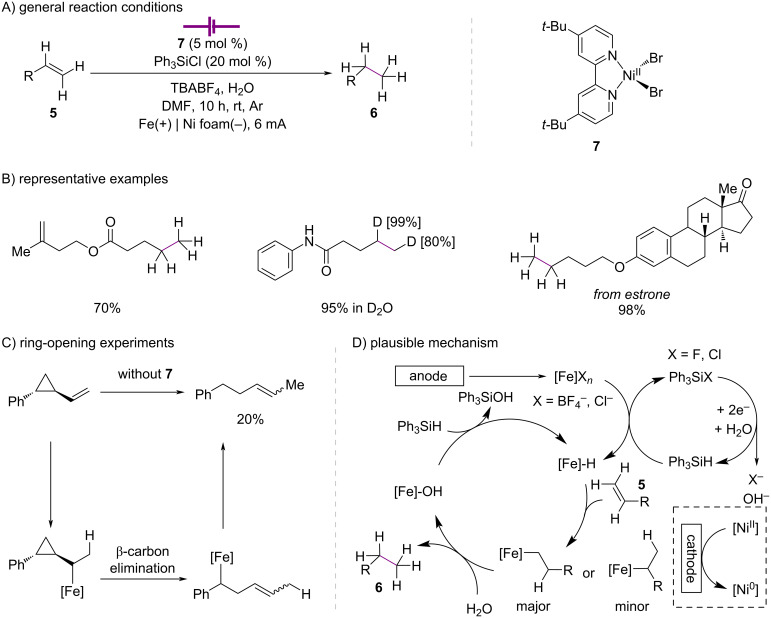
Electrochemical reduction of unactivated alkenes via iron catalysis.

In 2024, the Qiu group reported an electroreductive hydrogenation and deuteration of unactivated alkenes using galvanostatic electrolysis in an undivided cell, in which a catalytically active Fe–H species is generated in situ via a silane intermediate under electroreductive conditions (Scheme 2A) [89]. In this work, the authors highlight a conceptually important strategy that enables the use of H_2_O as a sustainable hydrogen source through a silane-mediated hydrogen-transfer pathway, thereby circumventing the direct use of water in hydrogen-transfer processes. Notably, the addition of a catalytic amount of a nickel complex was found to improve the reaction efficiency, which is attributed to the electroreductive generation of a Ni(0) species. In the presence of the in situ generated silane, a Ni–H species is proposed to form, although its involvement remains to be fully established [90]. Substrate scope studies demonstrated that not only terminal alkenes bearing free carboxylic acid or hydroxy groups, but also substrates containing multiple C=C bonds, undergo highly selective reduction at monosubstituted terminal alkene sites. These results highlight the broad functional-group compatibility and excellent chemoselectivity of the system (Scheme 2B).

Moreover, replacement of H_2_O with D_2_O enabled efficient electroreductive deuteration, and the successful late-stage electrohydrogenative functionalization of structurally complex molecules and drug derivatives further underscored the practical applicability of this strategy in medicinal chemistry contexts. To probe the involvement of catalytically relevant Fe–H species, radical probe substrates were subjected to the electroreductive conditions in the absence of the nickel complex. Under these conditions, both a ring-opening product and a hydrogenation product were detected by NMR spectroscopy. These observations provide compelling experimental evidence that Fe–H species, formed in situ through the combination of anodic iron salts and electrochemically generated silyl hydrides, actively participate in the reaction pathway (Scheme 2C) [91]. The authors proposed that the ring-opening product arises from β-carbon elimination of an intermediate generated via migratory insertion, followed by protonation to furnish the final product (Scheme 2D).

### Cobalt

#### Alkynes to alkenes

Cobalt readily operates within open-shell and radical manifolds [92–93], rendering it particularly effective for HAT-based reductive transformations.

**Scheme 3:**
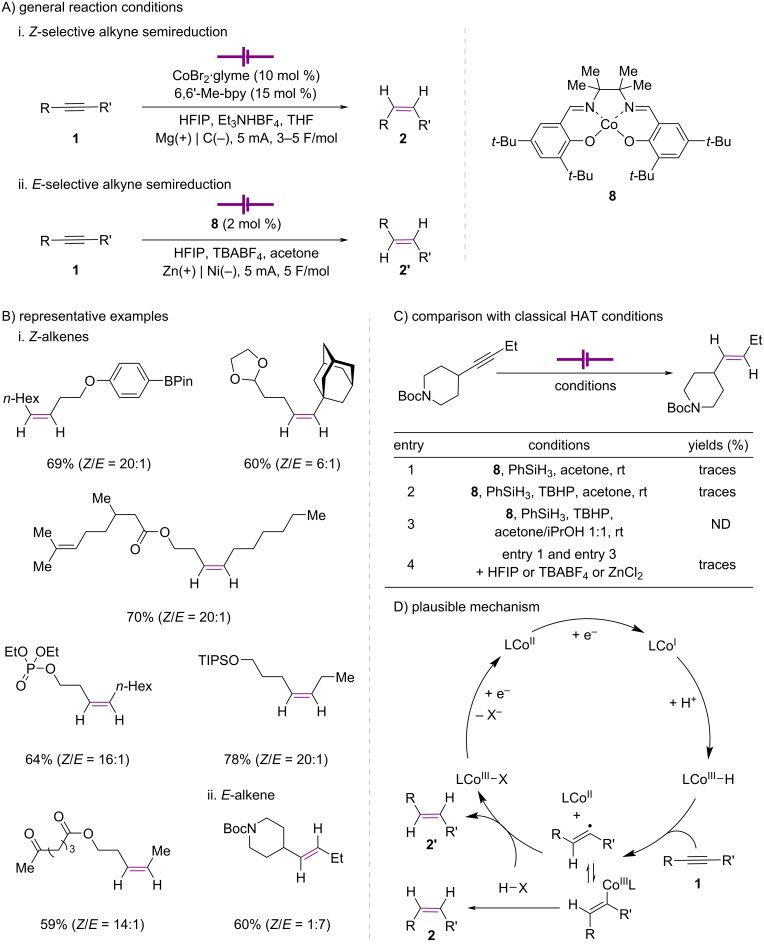
Cobalt-catalyzed e-HAT for the reduction of alkynes.

In 2022, the Baran group reported a ligand-controlled *E*- or *Z*-selective alkyne semireduction using cobalt catalysis under electrochemical HAT conditions (Scheme 3A) [94]. Notably, this study highlights a fundamentally distinct approach to HAT reactivity by replacing conventional stoichiometric reductants with an electrochemical platform, thereby enabling tunable reactivity and enhanced chemoselectivity that are difficult to achieve under classical HAT conditions. Substrates featuring a quaternary carbon adjacent to the alkyne exhibited diminished *E*/*Z* selectivity, which was tentatively attributed to increased steric hindrance surrounding the putative cobalt–alkene intermediate. Interestingly, alkynes bearing alkene moieties underwent selective reduction of the alkyne unit without perturbing the olefin, thereby demonstrating the high level of chemoselectivity of the reaction. Notably, the electrocatalytic HAT platform exhibits a markedly expanded substrate scope, tolerating functionalities such as amino alcohol motifs, benzyl-protected acids, and thioethers without competitive reduction. As a further demonstration of the tunability of e-HAT, the challenging *trans*-selective alkyne semireduction was also realized under modified electrochemical conditions (Scheme 3B, ii).

In contrast, analogous experiments conducted under conventional silane-based HAT conditions using the same cobalt complex **8** failed to reproduce this reactivity, underscoring the unique feature of the electrochemical platform (Scheme 3C). Cyclic voltammetry analysis of the *Z-*selective alkyne semireduction system indicated that protonation of low-valent cobalt species leads to the formation of a Co(III)–H intermediate [95], which then reacts with the alkyne to regenerate the monoligated Co(II) catalyst (Scheme 3D). DFT calculations further support that proton delivery from HFIP to the cobalt–alkyne adduct proceeds along an energetically viable pathway with a notably low barrier, consistent with a concerted mechanism. These findings collectively delineate the proposed *Z*-selective reduction catalytic cycle. Moreover, bond dissociation energy analysis revealed that the relatively weak Co–H (42.4 kcal mol^−1^) and Co–C (37.8 kcal mol^−1^) bonds in the Co–salen complex disfavor the formation of a persistent vinyl–cobalt σ-bond intermediate. Consequently, the resulting vinyl radical is more appropriately described as undergoing cage escape, thereby favoring a radical-type pathway rather than an organometallic sequence involving migratory insertion and β-hydride elimination, which ultimately accounts for the observed preference for *E*-selective alkyne reduction [96]. In addition to the alkyne reduction process, the same e-HAT platform could be extended to alkene isomerization and reduction as well.

**Scheme 4:**
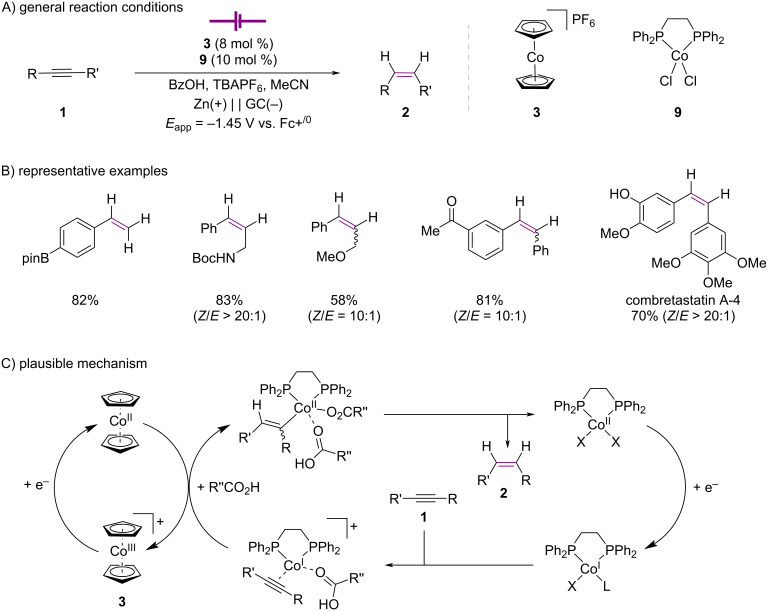
Dual cobalt catalyses for *Z*-selective semihydrogenation of alkynes.

In 2026, the Derosa group reported a voltage-controlled tandem electrocatalytic *Z*-selective semihydrogenation, in which cobaltocene operates as a cobalt redox mediator **3** in concert with a cobalt catalyst **9** (Scheme 4A) [97]. This strategy enables the selective hydrogenation of activated alkynes to *cis*-alkenes while exhibiting remarkably broad functional group tolerance, even in the presence of readily reducible functionalities (Scheme 4B). Also, the use of a redox mediator allows operation at a milder applied potential, thereby suppressing overreduction pathways and competitive hydrogen evolution while maintaining high catalytic efficiency. Notably, the transformation proceeds with exceptionally high Faradaic efficiency (up to ≈99%), indicative of highly effective suppression of competing hydrogen evolution under mediator-governed electrochemical conditions.

Mechanistic interrogation by cyclic voltammetry revealed two principal redox couples for the cobalt complex **9**, corresponding to the Co(II/I) couple at −1.0 V vs Fc^+/0^ and the Co(I/0) couple at −1.7 V vs Fc^+/0^. Direct controlled-potential electrolysis conducted at the more cathodic feature failed to deliver productive semihydrogenation, consistent with the hypothesis that excessive driving force diverts reactivity toward off-cycle reduction pathways and competitive hydrogen evolution. In sharp contrast, electrolysis performed at the mediator potential (≈ −1.45 V vs Fc^+/0^) enabled efficient catalytic turnover, demonstrating that productive reactivity emerges only under conditions in which both the redox mediator and the cobalt catalyst are simultaneously operative. Kinetic analysis revealed positive dependence on the concentrations of acid, alkyne, mediator, and cobalt catalyst, supporting a mechanistic regime in which substrate and acid binding equilibria precede a kinetically relevant proton/electron delivery event (Scheme 4C). DFT calculations proved a hydride-free, multisite proton-coupled electron transfer (MS-PCET) manifold [66–67,98], in which Cp_2_Co mediates electron transfer to an acid- and alkyne-associated cobalt intermediate, followed by acid-induced preorganization that enables inner-sphere proton-transfer steps and thereby enforces *Z*-selectivity.

Notably, electrochemical reduction further offers a compelling platform for selective deuterium incorporation, as isotopic labeling can be achieved directly from simple deuterium sources under precisely controlled redox conditions.

**Scheme 5:**
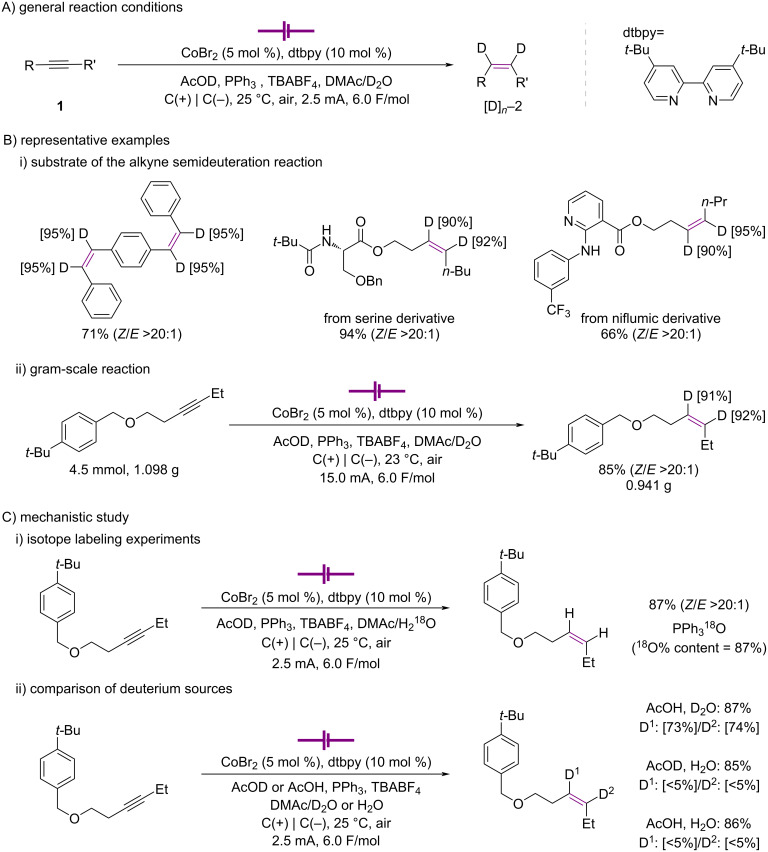
Electrochemical cobalt-catalyzed semideuteration of alkynes.

In 2025, the Fu group reported an electrochemical cobalt-catalyzed *Z*-selective semideuteration of alkynes (Scheme 5A) [99]. This protocol employs inexpensive cobalt salts as catalysts and utilizes D_2_O and AcOD as practical and efficient deuterium sources. The reaction exhibits a broad substrate scope, high efficiency, excellent functional-group tolerance, and consistently high levels of deuterium incorporation (Scheme 5Bi) [100–101]. Notably, the transformation proceeds efficiently with derivatives of biologically active molecules, such as serine and niflumic acid, which contain a wide array of functional groups. Furthermore, the reaction was successfully performed on a gram scale without erosion of either selectivity or efficiency (Scheme 5B, ii). Control experiments suggest that deuterium incorporation arises from both AcOD and D_2_O, rather than a single deuterium source (Scheme 5C) [102]. Cyclic voltammetry studies showed that, in the absence of dtbpy, only the Co(II)/Co(I) redox couple is accessible, whereas coordination of dtbpy enables electrochemical access to the Co(I)/Co(0) couple. Upon addition of AcOD, either alone or in combination with alkyne and D_2_O, the formation of deuterated low-valent cobalt species is suggested, which are proposed to give rise to catalytically relevant Co–D intermediates [94].

Kinetic analysis further revealed that, at a current of 2.5 mA, the reaction displays zeroth order dependence on alkyne, cobalt catalyst, and AcOD concentrations, whereas at a higher current of 7.5 mA, the rate becomes first-order in cobalt catalyst while remaining zero order in both alkyne and AcOD. Collectively, these observations indicate that the rate-determining regime is governed by the interplay between applied current and catalyst loading, highlighting the intrinsically electrochemical nature of turnover control in this system.

**Scheme 6:**
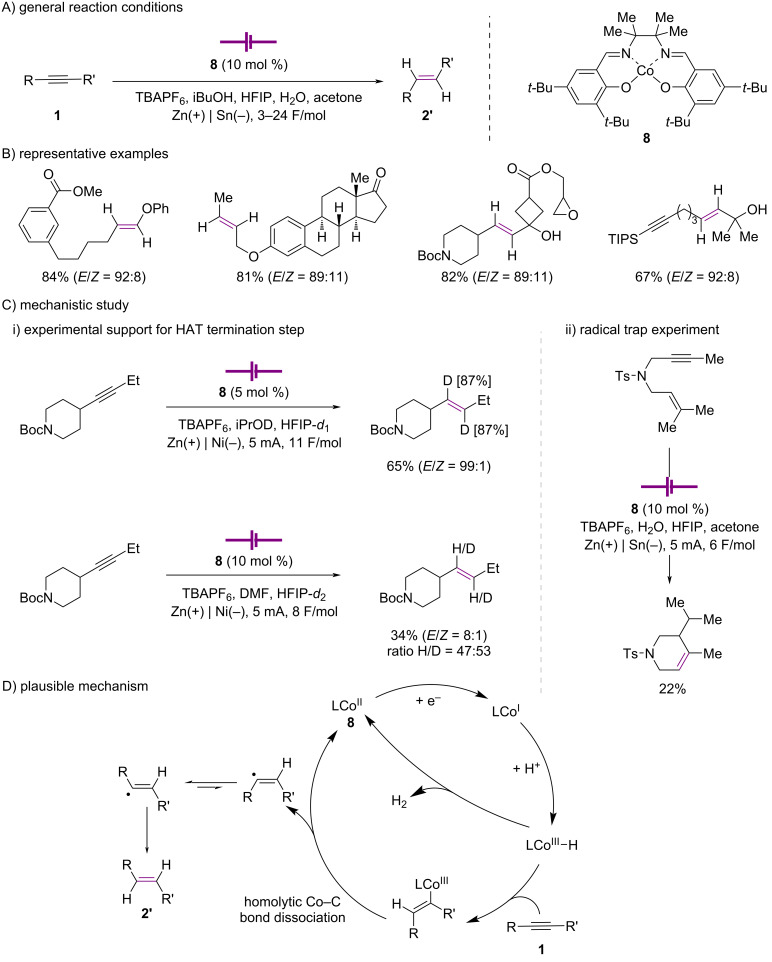
Electrochemical cobalt catalysis for *E*-selective semihydrogenation of alkynes.

In 2025, the Gnaim group further developed a *trans*-selective alkyne-reduction strategy based on a cobalt hydride radical-mediated pathway operating under one-electron logic, building upon the conceptual framework previously established in Baran’s study (Scheme 6A) [103]. Redox-sensitive functionalities such as esters, ketones, epoxides, nitriles, and arylboronic esters remained intact, and both electron-rich and electron-poor arenes were fully compatible with the reaction conditions (Scheme 6B). Importantly, this strategy enables the *trans*-selective semireduction of unactivated internal alkynes, addressing a limitation in previous methods that typically rely on electronically biased substrates. Moreover, simple substitution of the proton source with HFIP-*d*_1_ or iPrOD-*d*_1_ enabled efficient *trans*-selective deuteration of alkynes (Scheme 6C, i). An intramolecular radical trapping experiment employing enynes further corroborated the involvement of a vinyl radical intermediate, as evidenced by the formation of cyclized products via C–C bond formation followed by hydrogen abstraction (Scheme 6C, ii). Mechanistic studies revealed that cathodic reduction of Co(II) generates a Co(I) species, which is subsequently protonated by HFIP to form a cobalt hydride intermediate that engages with the alkyne substrate. Cyclic voltammetry supports the generation of Co–H species [60], while UV–vis spectroelectrochemical analysis revealed the emergence of new absorption features upon alkyne addition, consistent with the formation of a vinyl–Co(III) intermediate. DFT calculations further demonstrated that variation of the salen ligand framework modulates the Co–C bond-dissociation energy (BDE) of the vinyl–cobalt intermediate. Notably, decreasing Co–C BDEs correlate with an increased proportion of *trans*-alkene formation. This computational trend is consistent with the stereochemical rationale proposed in Baran’s study [94], in which facile homolytic Co–C bond cleavage was implicated in governing *trans*-selective alkene formation (Scheme 6D).

#### Alkenes to alkanes

**Scheme 7:**
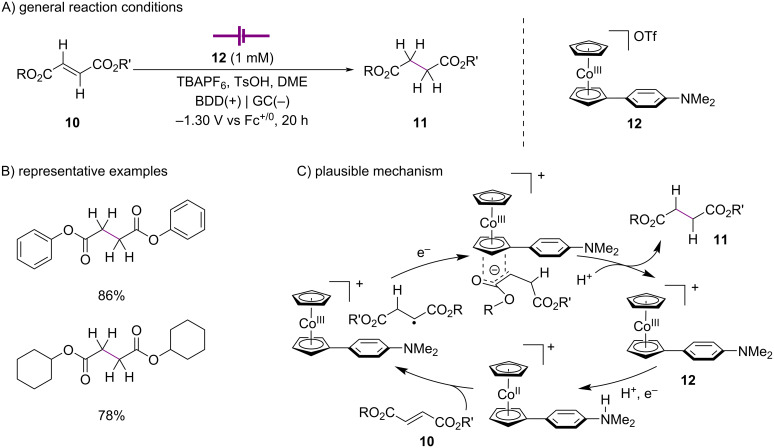
Concerted proton–electron transfer-driven electrocatalytic reduction of alkenes.

In 2021, the Peters group introduced a molecular design strategy that spatially separates the redox-active and proton-responsive sites within a CPET mediator, enabling controlled proton–electron delivery while minimizing undesired hydrogen evolution (Scheme 7A) [104]. Specifically, they developed a cobalt-derived redox catalyst **12** by incorporating an *N*,*N*-dimethylaniline Brønsted base into a cobaltocenium framework. To evaluate whether this cobalt-based mediator could facilitate net delivery of two protons and two electrons, formally equivalent to two hydrogen atoms, the authors selected fumarate esters as model substrates, as these species typically undergo oligomerization and competitive hydrogen evolution under conventional electrode-mediated reduction conditions (Scheme 7B).

In subsequent controlled-potential coulometry (CPC) experiments conducted at −1.30 V vs Fc^+/0^, the desired succinic acid derivatives were obtained. Importantly, this efficient conversion was observed only in the presence of the mediator; in its absence, product yields were markedly diminished and substantial amounts of starting material remained unreacted. According to the proposed mechanism, the reaction starts with an initial CPET event to the fumarate ester, generating a radical intermediate that is rapidly converted into a succinyl anion via a fast electron-transfer step. This anionic species subsequently engages with the Co(III) mediator to form a bound intermediate, thereby exhibiting co-catalytic behavior through direct interaction with the reaction intermediate, from which protonation releases the final product while regenerating the active mediator. Collectively, these observations support the operation of an ECEC-type pathway and provide strong mechanistic evidence that fumarate reduction proceeds most efficiently through a CPET-mediated manifold (Scheme 7C).

**Scheme 8:**
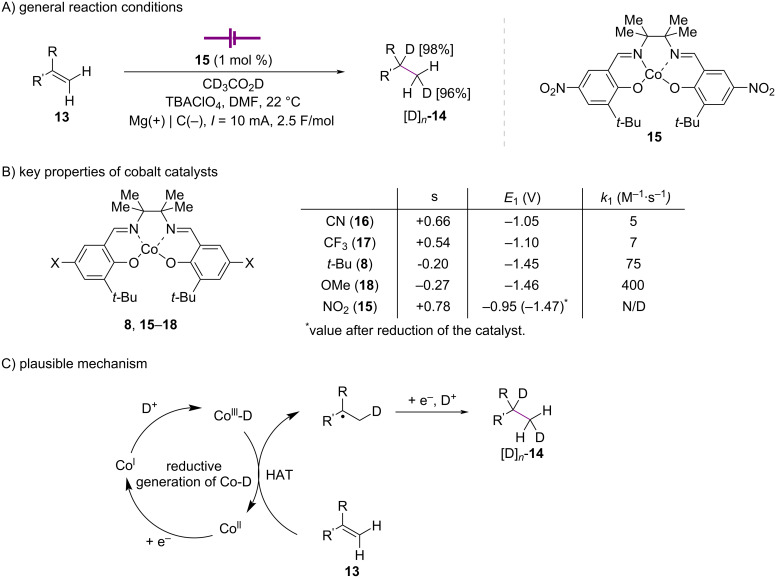
Electrochemical deuteration of alkenes via HAT using Co–H.

In 2022, the Lin group introduced a reductive strategy for the generation of Co–H species and demonstrated its applicability to the electroreductive deuteration of alkenes (Scheme 8A) [105]. To elucidate both the formation of Co(III)–H intermediates and their reactivity toward alkene substrates, the authors systematically investigated a series of cobalt salen complexes bearing electronically diverse ligand frameworks using complementary electrochemical and synthetic approaches. Rotating disk electrode (RDE) voltammetry revealed that hydrogen evolution reactivity is strongly dependent on the electronic properties of the ligand environment (Scheme 8B). Notably, this electronic modulation is directly reflected in the rate constant for Co(III)–H formation. Catalysts bearing electron-withdrawing substituents exhibit relatively low rate constants (5–7 M^−1^·s^−1^), whereas those incorporating electron-donating groups display substantially enhanced rates (75–400 M^−1^·s^−1^). These results strongly indicate that an electron-rich ligand environment facilitates Co(III)–H bond formation and, consequently, imparts increased reactivity in both hydrogen evolution and HAT processes (Scheme 8C).

### Nickel

#### Alkynes to alkenes

Nickel is an earth-abundant and versatile transition metal whose rich redox chemistry has enabled diverse catalytic transformations [106–109], rendering it an attractive platform for electrochemical reductive chemical transformations [110].

**Scheme 9:**
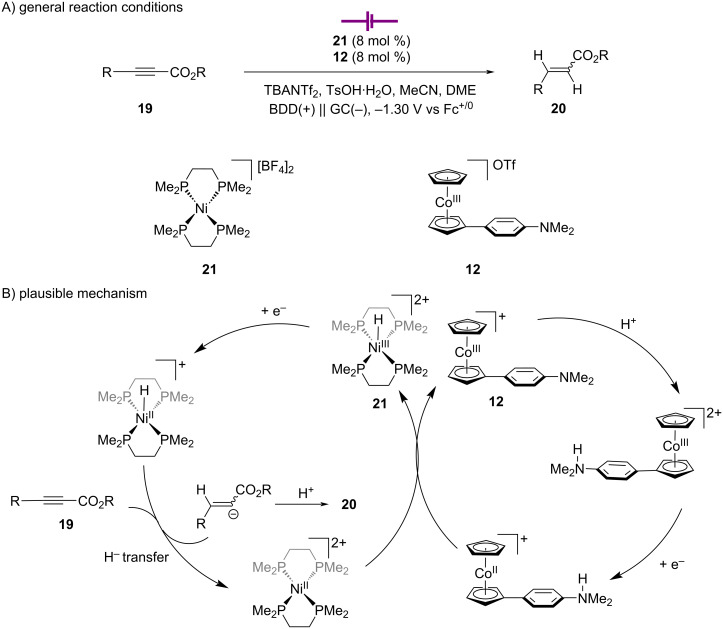
Electrocatalytic reduction of alkynes by hydride transfer via PCET.

In 2022, Peters and co-workers demonstrated that a nickel-based electrocatalyst for hydrogen evolution reactions (HER) can be repurposed as a hydride-delivery agent through incorporation of a cobalt-based PCET mediator under constant potential electrolysis with a divided cell setup (Scheme 9A) [111]. In certain established electroreductive nickel systems, such as the DuBois-type Ni–H complexes [112–114], hydride formation occurs at potentials coincident with HER or leads to undesired overreduction, both of which can suppress the desired substrate reduction. By contrast, the introduction of a cobaltocene-derived PCET mediator enables access to a Ni(III)–H intermediate at more anodic potentials, effectively achieving hydride generation from the Ni(II/0) redox process.

Under these tandem conditions, selective semihydrogenation is achieved with 63% yield at −1.30 V. Notably, product selectivity is highly sensitive to the applied potential, more cathodic conditions (−1.75 V) lead to increased HER, highlighting that precise potential control governs the competition between hydride transfer and proton reduction. Kinetic analysis reveals first-order dependence on both Ni and Co catalysts, with zeroth-order dependence on substrate and acid concentration, alongside a significant kinetic isotope effect (KIE ≈ 5.1), indicating that the PCET step is rate-determining. Based on this analysis, the authors proposed a PCET-mediated mechanism in which a rate-determining hydrogen-atom transfer from the redox mediator to Ni(II) generates a Ni(III)–H intermediate, which is subsequently reduced to Ni(II)–H and undergoes hydride transfer to the substrate, followed by protonation to yield the hydrogenated product (Scheme 9B).

**Scheme 10:**
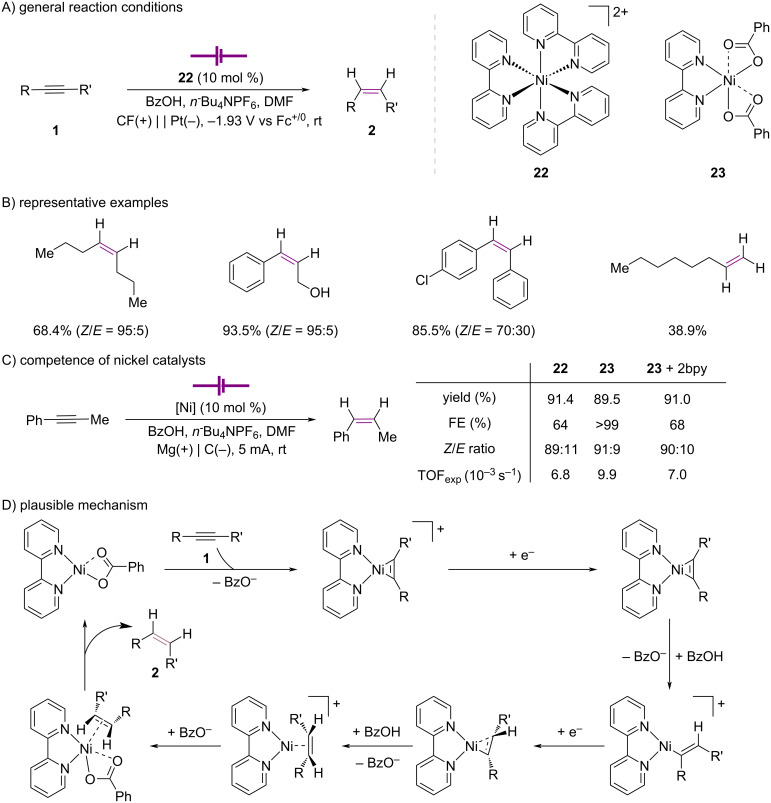
Ni–H-free electrocatalytic semihydrogenation of alkynes by nickel catalysis.

In 2022, the Kaeffer group reported an electrochemical nickel-catalyzed semihydrogenation of alkynes under constant potential electrolysis with divided cell setup, enabling selective alkene formation without the use of external H_2_ gas or stoichiometric chemical reductants (Scheme 10A) [115]. Under electrochemical control, the system exhibits high chemoselectivity and broad functional-group tolerance (Scheme 10B). A key aspect of this work is the hydride-free hydrogenation pathway, enabled by inner-sphere metal–substrate interactions involving organometallic intermediates, which allows a distinct electron- and proton-transfer manifold in alkyne reduction. Mechanistic interrogation by spectroelectrochemical techniques supported a hydride-free electron-transfer/proton-transfer (ET/PT) manifold [116–117], in which alkyne coordination to reduced nickel species generates a nickelacyclopropene intermediate [118–119] that undergoes two sequential protonation events to afford the *Z*-selective alkene product. In their subsequent study in 2023, isolation of this key intermediate provided direct experimental support for the proposed reaction pathway [120]. Also, a series of control experiments revealed that complex **23** is a markedly more competent electrocatalyst, delivering enhanced faradaic efficiency and turnover frequency (Scheme 10C). On the basis of cyclic voltammetry combined with DFT calculations, EECC-type pathways were ruled out as energetically unfavorable, whereas an ECEC-type sequence, defined by alternating electron-transfer and proton-transfer steps along on-cycle intermediates, was identified as the most consistent model for the observed reactivity and stereochemical outcome (Scheme 10D). Notably, this system was further examined for aldehyde substrates [121]. However, the electrochemically generated nickelacyclic intermediate requires subsequent stoichiometric protonation to afford the hydrogenation product, highlighting the limitations of directly extending the hydride-free ET/PT manifold to C=O bond reduction.

**Scheme 11:**
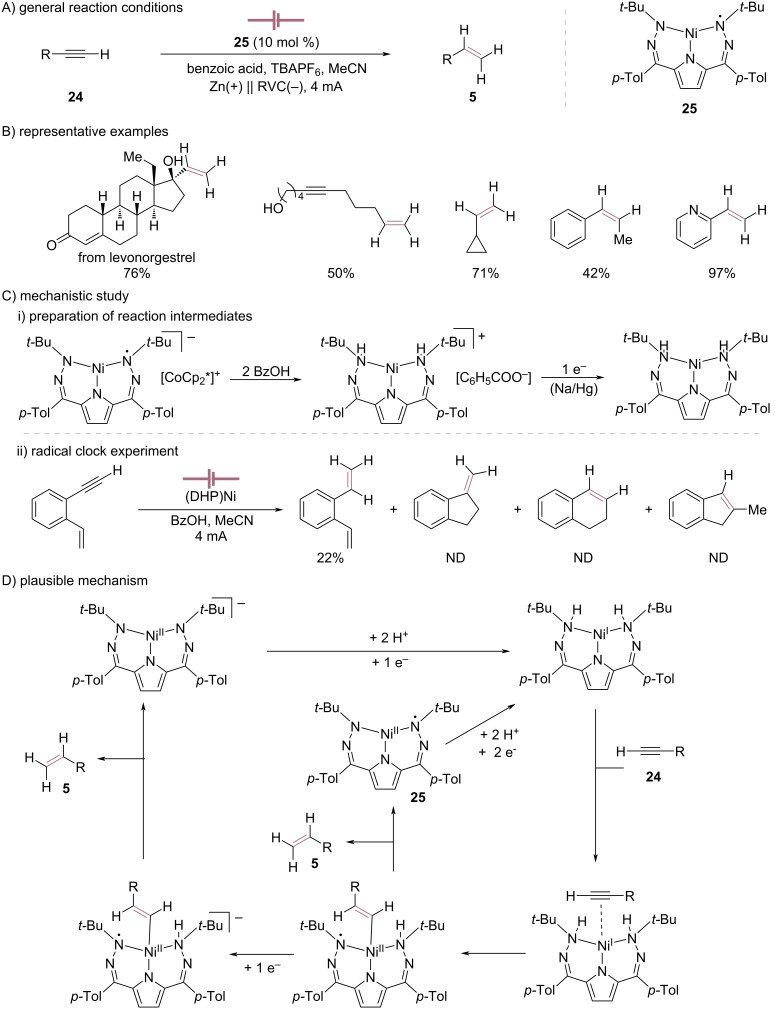
Ligand-based proton and electron transfer enabling electrocatalytic semihydrogenation of alkynes.

In 2024, the Anderson group reported an electrocatalytic semihydrogenation of alkynes [122] enabled by ligand-based storage and delivery of an H_2_ equivalent on a dihydrazonopyrrole (DHP) scaffold (Scheme 11A) [123–124]. The DHP–Ni system exhibits pronounced chemoselectivity for terminal alkynes while efficiently discriminating against their internal counterparts while the catalytic manifold can also be extended to internal alkynes under appropriately tuned conditions (Scheme 11B). A high level of functional group tolerance, with no detectable overreduction or oligomerization, further enables productive late-stage semihydrogenation of structurally complex molecules. Cyclic voltammetric analysis revealed a zeroth-order dependence on acid concentration, strongly supporting an intramolecular HAT event as the kinetically controlling step. In situ generation and characterization of (DHP)Ni intermediates are consistent with the involvement of a reduced, formal Ni(I) species as a key catalytic entry point (Scheme 11C, i). Moreover, radical-probe experiments failed to produce cyclized products, arguing against the presence of long-lived radical intermediates and instead supporting a concerted HAT manifold (Scheme 11C, ii). Notably, this mechanism is hydride-free, operating through reversible ligand-based N–H storage and direct hydrogen-atom shuttling from the DHP framework to the alkyne substrate (Scheme 11D). It is noteworthy that a subsequent study from this group could further expand this catalytic platform to Markovnikov-selective electrocatalytic hydroalkylation, thereby demonstrating the broader applicability of ligand-based H_2_ equivalent storage for controlling regioselective C–C bond formation beyond semihydrogenation chemistry [125].

**Scheme 12:**
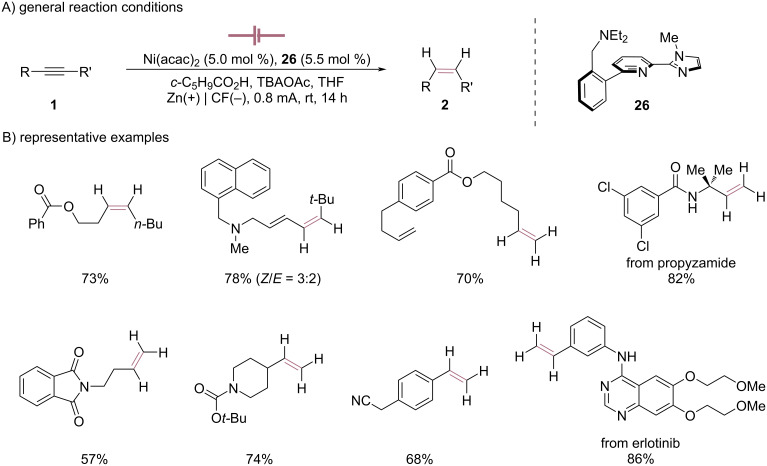
Proton-modulated electrochemical Ni–H formation for the hydrogenation of alkynes.

In 2025, the Wang group reported a nickel electrocatalytic system featuring a bifunctional ligand equipped with a pendant amine, specifically designed to promote Ni–H formation while simultaneously suppressing undesired side reactions, most notably hydrogen evolution reactions (HER) (Scheme 12A) [126]. This catalytic platform enabled selective semihydrogenation of both internal and terminal alkynes, including unactivated substrates, with broad tolerance toward heterocycles and alcohol functionalities (Scheme 12B). Interestingly, in enyne substrates, the alkene moiety remained intact while the alkyne underwent selective semihydrogenation, highlighting the chemoselectivity of the process.

**Scheme 13:**
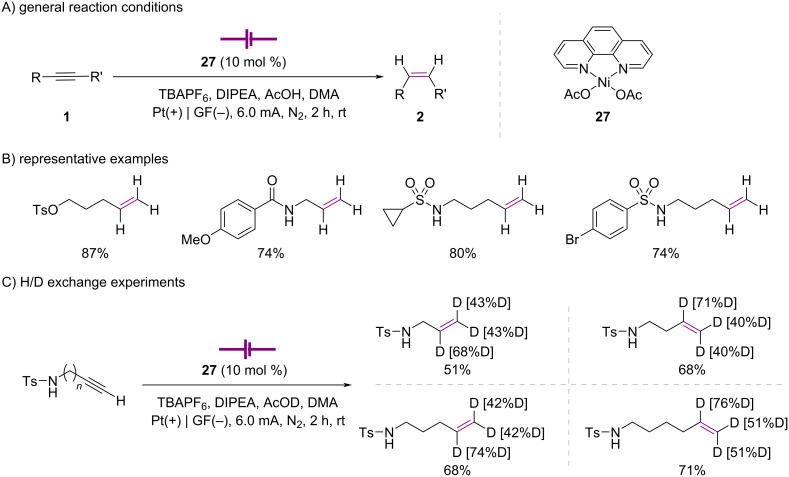
Nickel-catalyzed electrochemical semireduction of terminal alkynes.

Very recently, the Choi group reported a chemoselective electroreductive transformation of terminal alkynes to alkenes (Scheme 13A) [127]. In this reaction, a nickel(II) phenanthroline complex was employed in the presence of acetic acid as the proton source. A broad range of functional groups, including those typically sensitive under hydrogenation conditions, such as bromides and nitriles, were well tolerated, affording the corresponding alkenes with high selectivity (Scheme 13B). Notably, substrates bearing sulfonamides, sulfonates, *N*,*N*-disubstituted tosylamides, and secondary amides also underwent efficient and selective semireduction. Mechanistic investigations using deuterated acetic acid revealed that deuterium incorporation occurs exclusively at the vinylic positions of the terminal alkene, with no evidence for internal alkene formation (Scheme 13C). Consequently, these observations indicate that the semireduction pathway proceeds without the involvement of a nickel hydride species, instead favoring a proton-transfer mechanism.

#### Alkynes to alkanes

**Scheme 14:**
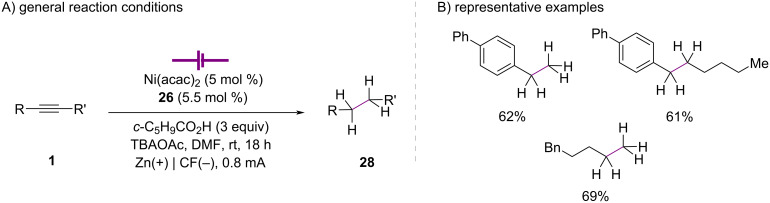
Full hydrogenation of alkynes via nickel hydride electrocatalysis.

The Wang group also demonstrated that a full alkyne reduction protocol can also be achieved under the same reaction conditions to those shown in Scheme 12 (Scheme 14A) [126]. They emphasized that product selectivity can be precisely modulated through simple operational parameters, the stoichiometry of the proton source, and the reaction time. Within this tunable platform, selective full hydrogenation was achieved across a diverse range of alkyne substrates, including terminal, internal, and aliphatic alkynes (Scheme 14B). Notably, in the intermediate regime between these two operational windows, a gradual erosion of product selectivity was observed, reflecting a continuous rather than discrete transition between semihydrogenation and complete reduction pathways.

**Scheme 15:**
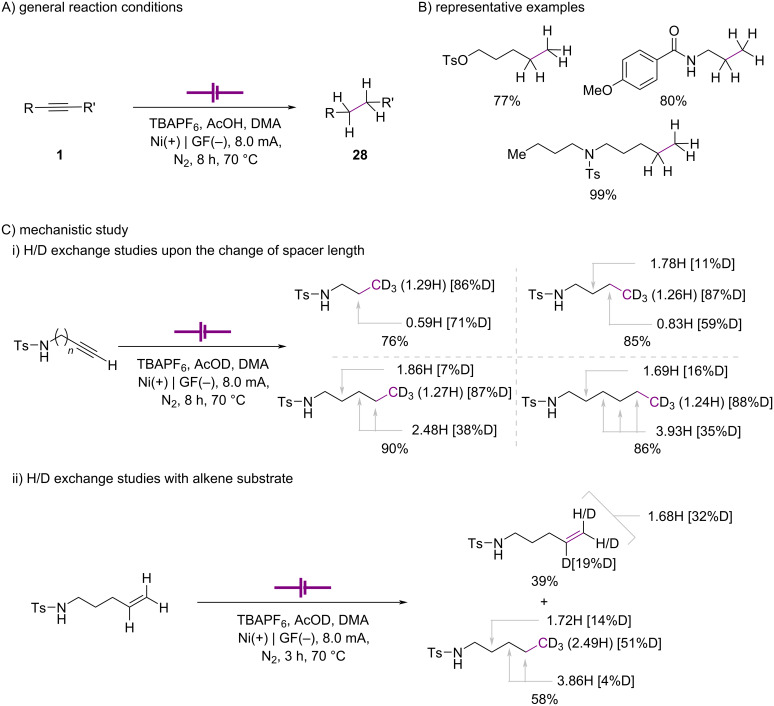
Ni–H-mediated electrochemical reduction of alkynes to alkanes.

The divergent electroreductive system developed by the Choi group could be further extended to the selective reduction of terminal alkynes to alkanes (Scheme 15A) [127]. In contrast to the semireduction conditions, the full reduction was achieved using a nickel sacrificial anode in the presence of acetic acid as the proton source. Under the optimized full reduction conditions, the reaction proceeded with high chemoselectivity across a broad range of substrates, demonstrating excellent functional-group tolerance (Scheme 15B). Mechanistic interrogation using deuterated acetic acid revealed that, unlike the semireduction pathway, deuterium incorporation occurs not only at the olefinic position but also along the alkyl chain (Scheme 15C, i). These observations indicate the involvement of a nickel hydride species and subsequent chain-walking processes. Furthermore, when the reaction was performed using an alkene substrate, deuterium incorporation was also observed in the recovered alkene, collectively supporting the operation of a reversible hydronickelation process with nondissociative nickel migration (Scheme 15C, ii).

#### Alkenes to alkanes

**Scheme 16:**
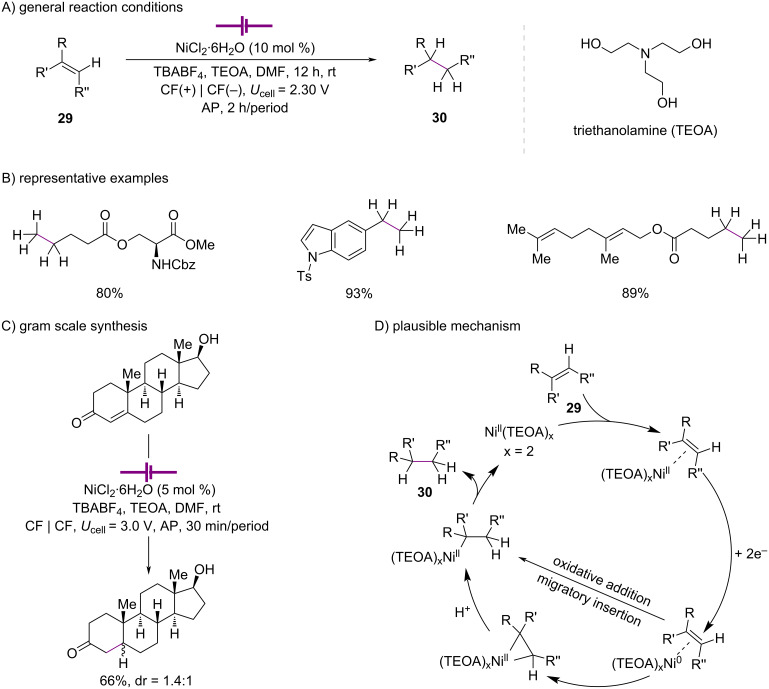
Electrochemical nickel-catalyzed hydrogenation of alkenes.

In 2024, the Fu group reported an electrochemical protocol for alkene reduction to alkanes with a strategy of an alternating polarity (AP) [128–130] to fundamentally suppress deleterious side reactions associated with cathodic metal deposition (Scheme 16A) [131]. Through this reaction design, an efficient and selective alkene-to-alkane reduction was achieved. Substrate scope studies demonstrated broad applicability across structurally diverse olefins, and the method proved readily amenable to late-stage functionalization of complex molecules (Scheme 16B).

The broad functional-group tolerance observed in this system was attributed to the ability of the nickel catalyst to effectively inhibit direct cathodic reduction of olefins. Furthermore, the electrochemical hydrogenation was successfully conducted on a 10 mmol scale, delivering the desired alkane products with high efficiency (Scheme 16C). On the basis of mechanistic analysis, the authors proposed that pre-coordination of either triethanolamine (TEOA) or the alkene substrate to the nickel center enables formation of an alkyl–Ni(II) species, either through generation of a nickelacyclopropane intermediate or via facile oxidative addition followed by migratory insertion (Scheme 13D).

**Scheme 17:**
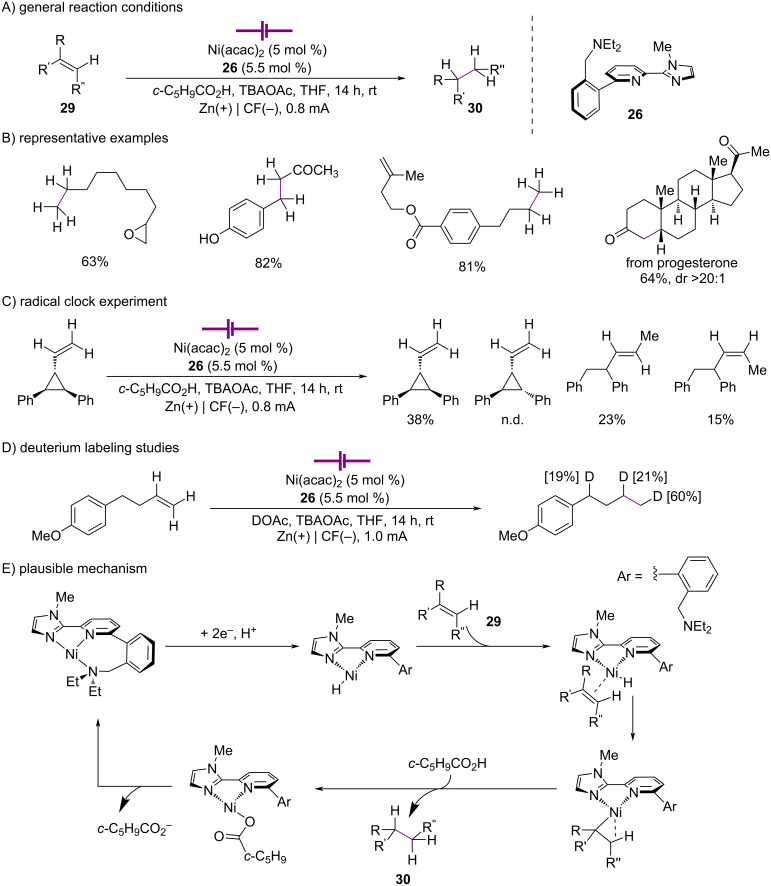
Electrochemical Ni–H formation and hydrogenation of alkenes using a bifunctional ligand.

Building on the concepts discussed above (Scheme 12 and Scheme 14), the Wang group further expanded the reaction scope to the complete reduction of alkenes to single C–C bonds (Scheme 17A) [126]. This method exhibits broad substrate generality with a wide range of functional groups that remain fully intact under the reaction conditions (Scheme 17B). Both activated and unactivated olefins, including internal and terminal substrates, were efficiently hydrogenated, and the protocol was successfully applied to natural products and pharmaceutical derivatives. Particularly striking is the hydrogenation of progesterone, which proceeds with exclusive formation of a single diastereomer. Radical clock experiments provided compelling experimental evidence that the reaction does not proceed through a radical pathway (Scheme 17C). In addition, deuterium-labeling studies using deuterated proton sources revealed selective incorporation of deuterium at the benzylic position of the product, supporting the involvement of a Ni–H intermediate operating through a chain-walking mechanism as the modus operandi of this transformation (Scheme 17D) [132]. DFT calculations further substantiated the feasibility of Ni–H generation and indicated that, following migratory insertion, the resulting alkyl–Ni(I) complex undergoes protonation by acid to furnish the hydrogenated product (Scheme 17E). It is noteworthy that the nature of the proton source fundamentally alters the reaction pathway, with distinct mechanistic regimes arising from differences in protonation mode, hydrogen-bonding interactions, and their impact on Ni–H insertion and β-hydride elimination steps.

**Scheme 18:**
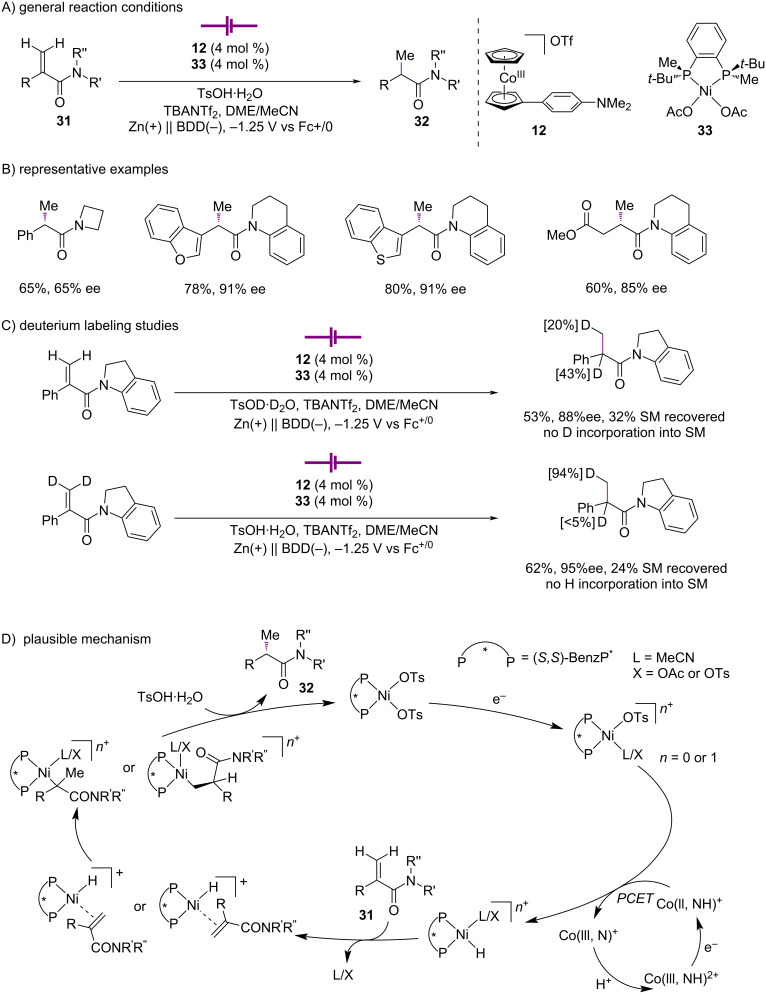
Nickel hydride electrocatalysis for the hydrogenation of alkenes.

The Peters group demonstrated that incorporation of a cobaltocene-derived mediator enables generation of a Ni(II)–H intermediate at an applied potential anodic to the Ni(II/I)–H redox couple, thereby allowing the intermediate to engage with alkene substrates rather than undergoing rapid hydrogen evolution [111,133]. On the basis of this concept, the group subsequently leveraged their previously established Ni/Co tandem electrochemical PCET (ePCET) platform together with design principles from Ni-catalyzed asymmetric hydrofunctionalization to extend mediator-assisted electrocatalysis to the asymmetric hydrogenation of C–C double bonds (Scheme 18A) [134]. The transformation accommodates a broad range of tertiary amides and maintains high yields and enantioselectivities across substrates bearing diverse electronic and steric profiles (Scheme 18B). These results clearly demonstrate that the cobalt mediator can be productively integrated with a chiral nickel cocatalyst and structurally varied substrates to enable efficient, stereocontrolled hydrogenation under comparatively mild, fixed-potential conditions. Mechanistic studies using deuterium-labeled substrates further indicated that hydride transfer to the α-carbon is reversible, while hydride transfer to the β-carbon proceeds with an effectively irreversible character (Scheme 18C). This divergence in reversibility depending on the insertion site provides experimental evidence that Ni–H insertion operates with distinct regioselective manifolds at the α- versus β-position. Collectively, these observations strongly support an operating principle in which electrochemical activation of the nickel(II) precatalyst is coupled to cobalt-based PCET mediation to generate and exploit a catalytically competent Ni–H species, or an equivalent hydrogen-delivery state, at a comparatively mild fixed potential, thereby suppressing competitive HER and enabling productive substrate hydrogenation (Scheme 18D).

## Conclusion

In this review, we have discussed that electrochemical reduction has matured into a powerful and conceptually distinct platform for the controlled transformation of unsaturated C–C bonds when combined with 3d transition-metal catalysis, including iron, cobalt, and nickel. Notably, across these metals, electrochemical potential and current function not merely as operational parameters but as decisive mechanistic selectors that govern catalyst speciation, regulate proton and electron delivery, and suppress unproductive pathways such as hydrogen evolution, thereby unlocking reactivity manifolds that are difficult to access under thermochemical conditions. Within this context, the reactivity of 3d transition-metal catalysts can be more precisely delineated in terms of metal-dependent engagement with distinct mechanistic regimes. In sharp contrast to classical expectations, iron systems typically operate under conditions in which the formation of stable metal hydride species is disfavored, thereby promoting hydride-free pathways or stepwise ET/PT processes involving substrate-bound intermediates. Notably, cobalt catalysts exhibit pronounced mechanistic flexibility, as they can access both metal hydride and hydride-free manifolds, with well-defined systems proceeding through Co–H intermediates that function as hydrogen-atom-transfer (HAT) donors, while alternative pathways engage multisite PCET or mediator-enabled processes. Nickel catalysts, on the other hand, more consistently favor inner-sphere organometallic reactivity, including migratory insertion and metallacycle formation, while retaining the capacity to operate through hydride transfer or hydride-free pathways depending on ligand environment and applied potential. Consequently, the selection of mechanistic mode is not intrinsic to a single pathway but is instead governed by the interplay between metal identity, ligand structure, and electrochemical conditions, thereby providing a unified framework for understanding reactivity trends across iron, cobalt, and nickel-catalyzed electrochemical hydrogenation of unsaturated C–C bonds. Consequently, electrochemical hydrogenation does not proceed through a single dominant pathway but instead emerges from the selective engagement of distinct mechanistic manifolds. Taken together, these advances establish 3d transition-metal electrocatalysis as a programmable platform for achieving precise and tunable hydrogenation of unsaturated C–C bonds. Based on these strategies, several promising directions can be also envisioned for further advancing the field. In particular, expansion to other 3d transition metals represents an important opportunity, as the diverse redox properties and coordination environments accessible across the 3d series may enable fundamentally new reactivity patterns and mechanistic regimes that remain unexplored [118,135–136]. Furthermore, extending these electrochemical platforms beyond hydrogenation toward more diverse reductive chemical transformations will significantly broaden their synthetic utility, enabling access to a wider range of bond constructions under mild and sustainable conditions [137–138].

## Data Availability

Data sharing is not applicable as no new data was generated or analyzed in this study.
